# Health benefits from moderate drinking: a critical review

**DOI:** 10.1186/1940-0640-10-S2-O2

**Published:** 2015-09-24

**Authors:** Sven Andreasson, Tanya Chikritzhs, Frida Dangardt, Harold Holder, Timothy Naimi, Tim Stockwell

**Affiliations:** 1 Karolinska Institutet - Department of Public Health Sciences. Stockholm; Sweden; 2 Curtin University - National Drug Research Institute, Perth, Western Australia; 3 Sahlgrenska Academy and University Hospital, The Queen Silvia Children's Hospital - Peadiatric Clinical Physiology, Goteborg; Sweden; 4 Prevention Research Center, Pacific Institute for Research and Evaluation, 180 Grand Ave., Suite 1200, Oakland, CA 94612, USA; 5 Boston Medical Center - Section on General Internal Medicine; Boston University Schools of Medicine and Public Health, USA; 6 Centre for Addictions Research of BC, University of Victoria; BC, Canada

## 

Although alcohol consumption is a leading cause of preventable death and social problems worldwide, many studies have found an association between low-dose consumption and reduced risk of cardiovascular (CVD) disease. Despite important limitations in the underlying research, this idea has been promoted extensively, used to argue against the adoption of effective alcohol policies and led some doctors to advise patients to drink for better health.

There are a number of grounds for scepticism about these claims. There have been no randomised studies of low-dose alcohol consumption with disease or death outcomes to confirm findings from non-randomised studies. There are many methodological problems with observational studies, most importantly confounding and misclassification. In contrast to observational studies, a recent large Mendelian randomisation study found that having a genetic disposition that causes less drinking was associated with a significantly reduced risk of coronary disease, even among those who consume modest amounts of alcohol. Recent research has also challenged some of the purported mechanisms for the protective effect of moderate drinking, eg the effect on and impact of blood lipids.

This paper will provide methodological critique of the scientific basis for the claim that low-dose alcohol confers health benefits. It is concluded that the protective effects of moderate drinking may be spurious. Governments should strengthen effective alcohol control policies to reduce alcohol-related deaths, social problems and economic costs. Physician advice to patients should focus on reducing consumption among current drinkers, and should discourage drinking initiation or increased consumption on the basis of health-related considerations.

